# Muscle Strength Profiles According to Kidney Dysfunction and Anemia Status in Korean Adults Aged ≥40 Years: A Nationwide Cross-Sectional Study

**DOI:** 10.3390/healthcare14183043

**Published:** 2026-09-16

**Authors:** Hyeeun Son, Ian Bak, Jungmin Lee, Su Jung Lee

**Affiliations:** 1 School of Nursing, Hallym University, Chuncheon 24252, Republic of Korea; nason0713@gmail.com (H.S.); ianalistairbak@gmail.com (I.B.); 2 School of Nursing, Research Institute of Nursing Science, Hallym University, Chuncheon 24252, Republic of Korea; j_lee0624@hallym.ac.kr

**Keywords:** renal insufficiency, anemia, hand strength, cross-sectional studies

## Abstract

**Background/Objectives:** Kidney dysfunction and anemia may each be associated with impaired muscle strength, but evidence on their concurrent presence is limited. We examined muscle strength according to kidney dysfunction and anemia status in Korean adults aged ≥40 years. **Methods:** This cross-sectional study used 2024 Korea National Health and Nutrition Examination Survey data. Kidney dysfunction was defined as estimated glomerular filtration rate < 60 mL/min/1.73 m^2^ or urinary albumin-to-creatinine ratio ≥ 30 mg/g; anemia as hemoglobin < 13 g/dL in men and <12 g/dL in women. Complex-sample logistic regression and general linear models analyzed 3649 participants in four groups. **Results:** Compared with participants with neither condition, the primary confounder-adjusted odds of low muscle strength were higher in the anemia-only group (adjusted odds ratio [aOR], 2.38; 95% confidence interval [CI], 1.57–3.61) and concurrent group (aOR, 3.31; 95% CI, 1.86–5.89), but not in the kidney dysfunction-only group (aOR, 1.42; 95% CI, 0.88–2.30). The concurrent group had the largest adjusted deficit in maximum handgrip strength (−2.51 kg; *p* < 0.001). Bonferroni-adjusted direct comparisons showed significantly poorer outcomes in the concurrent group than in the kidney dysfunction-only group, but not the anemia-only group. **Conclusions:** The concurrent presence of kidney dysfunction and anemia was associated with the least favorable muscle strength profile, particularly relative to kidney dysfunction alone; differences from anemia alone were not statistically significant after multiplicity correction.

## 1. Introduction

Kidney dysfunction may affect physical function, in addition to impairing renal filtration, with muscle weakness representing an important functional concern. Low muscle strength is a central feature of sarcopenia and is an important indicator of impaired physical function. The revised European consensus underscores low muscle strength as a principal characteristic of sarcopenia, with muscle quantity or quality used to confirm the diagnosis [1]. Similarly, the Asian Working Group for Sarcopenia places muscle strength at the center of sarcopenia assessment and recommends sex-specific handgrip strength thresholds for Asian populations [2]. Handgrip strength is widely used as a simple, objective, and noninvasive measure of muscle strength. Its relevance extends beyond sarcopenia, as lower handgrip strength has been associated with adverse health outcomes across the life course [3,4].

Evidence supports the relationship between kidney and muscle health. In a prospective UK Biobank study, participants in the highest sex-specific quartile of handgrip strength had a 28% lower risk of incident chronic kidney disease (CKD) than those in the lowest quartile (hazard ratio [HR], 0.72; 95% confidence interval [CI], 0.67–0.77) [5]. In the general United States population, each 1-kg increase in grip strength was associated with a 0.49-mg/g lower urinary albumin-to-creatinine ratio (UACR) after multivariable adjustment [6]. A global meta-analysis of patients with CKD reported sarcopenia in 24.5%, low muscle strength in 43.4%, and low muscle mass in 29.1% of patients, supporting the involvement of both muscle quantity and strength in CKD-related muscle impairment [7]. Sarcopenia and lower handgrip strength have also been associated with adverse clinical outcomes and mortality in CKD [8,9,10].

Several biological pathways are involved in the association between impaired kidney function and muscle strength. In CKD, multiple factors, including inflammation, oxidative stress, metabolic acidosis, uremic toxin accumulation, insulin resistance, and disturbances in muscle protein metabolism, may contribute to skeletal muscle catabolism and impaired muscle function [11,12]. Nevertheless, kidney dysfunction identified in a single examination does not equate to a confirmed diagnosis of CKD, which requires evidence of chronicity. In this study, we used reduced estimated glomerular filtration rate (eGFR) and albuminuria as markers of kidney dysfunction, rather than CKD-confirmatory factors.

Anemia is frequently accompanied by kidney dysfunction and may constitute an additional burden on muscle function. In CKD, anemia can result from reduced erythropoietin production, impaired iron availability, inflammation, and reduced erythrocyte survival [13]. Reduced hemoglobin concentrations may limit oxygen delivery to the skeletal muscles, potentially contributing to fatigue, reduced exercise capacity, and impaired muscle performance. In a nationwide study of older Korean adults, anemia was associated with greater odds of sarcopenia after multivariable adjustment (odds ratio [OR], 1.46; 95% CI, 1.06–1.99) [14]. Another Korean population-based study reported lower mean handgrip strength in participants with anemia, particularly among men (30.84 vs. 38.97 kg) [15]. Hemoglobin concentration has also been positively associated with grip strength after accounting for relevant clinical factors [16], and a meta-analysis reported lower hemoglobin concentrations among individuals with sarcopenia [17]. Prospectively, incident anemia was associated with incident sarcopenia (relative risk ratio, 3.64; 95% CI, 1.18–11.19), while persistent anemia was associated with persistent sarcopenia (relative risk ratio, 3.59; 95% CI, 1.14–11.27) [18].

Despite these findings, kidney dysfunction and anemia have been examined separately in relation to muscle health. Kidney dysfunction may contribute to impaired muscle function through metabolic and catabolic disturbances, whereas anemia may impose an additional functional burden through a reduced oxygen-carrying capacity. Because these two conditions frequently coexist, evaluating mutually exclusive groups can distinguish muscle strength profiles associated with kidney dysfunction alone, anemia alone, and their concurrent presence. Importantly, the less favorable profile in the concurrent group did not demonstrate a statistical interaction itself.

Therefore, this study examined the individual and concurrent associations of kidney dysfunction and anemia with low muscle strength and maximum handgrip strength among Korean adults aged ≥40 years using data from the 2024 Korea National Health and Nutrition Examination Survey (KNHANES). We hypothesized that participants with both kidney dysfunction and anemia would have lower maximum handgrip strength and higher odds of low muscle strength than those with either condition alone or neither condition. We also examined the interaction between kidney dysfunction and anemia.

## 2. Materials and Methods

### 2.1. Study Design and Participants

This cross-sectional study used data from the 2024 KNHANES, conducted by the Korea Disease Control and Prevention Agency. The KNHANES is a nationwide survey comprising health interviews, health examinations, and nutrition surveys, and uses a stratified, multistage, clustered probability sampling design to obtain a representative sample of the non-institutionalized Korean population.

**Figure 1:**
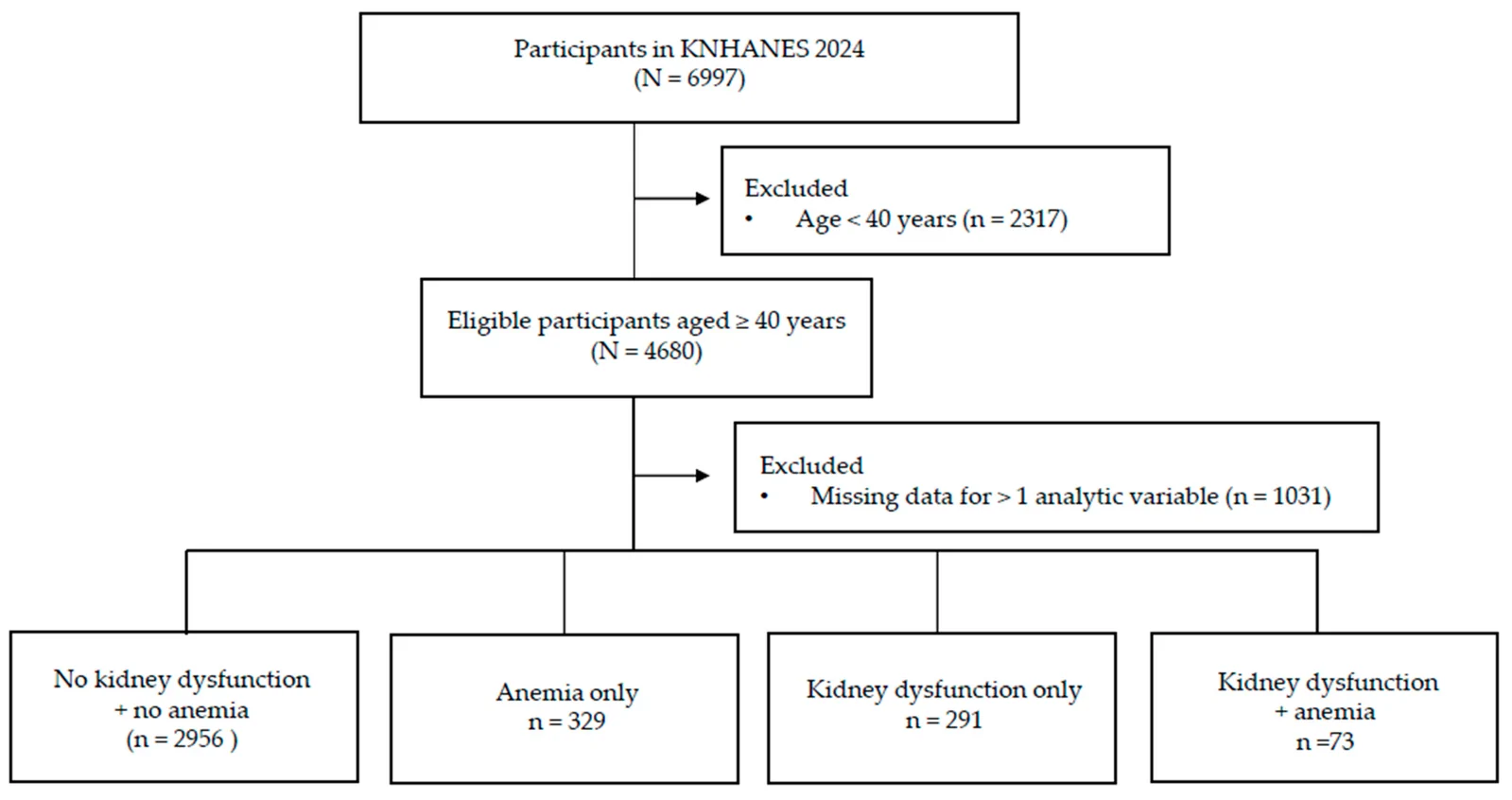
Flow diagram of participant selection.

Among the 6997 participants in KNHANES 2024, 2317 participants aged <40 years were excluded because handgrip strength was assessed only in participants aged ≥40 years. Of the 4680 eligible participants, 1031 (22%) had missing data for at least one analytical variable and were excluded from the complete case analysis. The final analytical sample comprised 3649 (78%) participants (Figure 1).

#### 2.1.1. Maximum Handgrip Strength and Low Muscle Strength

Handgrip strength was measured using a digital grip strength dynamometer (T.K.K. 5401; Takei Scientific Instruments Co., Ltd., Niigata, Japan). In the 2024 KNHANES, handgrip strength was measured twice for each hand. The highest value among the four measurements was defined as the maximum handgrip strength and was used in the analysis. Low muscle strength was defined according to the 2019 Asian Working Group for Sarcopenia criteria as a maximum handgrip strength < 28 kg in men and <18 kg in women [2].

#### 2.1.2. Kidney Dysfunction and Anemia

Kidney dysfunction was operationally classified using the eGFR and UACR. eGFR was calculated using the 2021 Chronic Kidney Disease Epidemiology Collaboration creatinine equation [19]. Albuminuria was defined as a UACR ≥ 30 mg/g. Kidney dysfunction was defined as an eGFR < 60 mL/min/1.73 m^2^ or a UACR ≥ 30 mg/g, corresponding to the Kidney Disease: Improving Global Outcomes G3a–G5 or A2–A3 categories [20]. Because eGFR and UACR were measured during a single examination, this classification represented kidney dysfunction at the time of the survey and did not establish a diagnosis of CKD.

Anemia was defined as a hemoglobin concentration < 13.0 g/dL in men and <12.0 g/dL in non-pregnant women, respectively [21]. Hemoglobin was measured using the cyanide-free hemoglobin spectrophotometric method with an automated hematology analyzer (XN-1000, Sysmex, Kobe, Japan).

Participants were classified into four mutually exclusive categories: (1) no kidney dysfunction or anemia, (2) anemia only, (3) kidney dysfunction only, and (4) both kidney dysfunction and anemia. Participants with neither condition constituted the reference group.

#### 2.1.3. Participant Characteristics

The participant characteristics included age, sex, body mass index (BMI), aerobic physical activity, diabetes mellitus, hypertension, myocardial infarction or angina, and high-sensitivity C-reactive protein (hs-CRP). BMI was calculated as weight in kilograms divided by height in meters squared (kg/m^2^). Meeting the aerobic physical activity recommendation was defined as engaging in at least 150 min of moderate-intensity activity, at least 75 min of vigorous-intensity activity, or an equivalent combination per week [22]. Diabetes mellitus was defined as a fasting plasma glucose level ≥ 126 mg/dL, glycated hemoglobin level ≥ 6.5%, a physician’s diagnosis of diabetes [23], or current use of glucose-lowering medication or insulin. Hypertension was defined as a systolic blood pressure ≥ 140 mmHg, a diastolic blood pressure ≥ 90 mmHg [24], or the current use of antihypertensive medication. Myocardial infarction or angina was defined based on a self-reported physician diagnosis of either condition. Because hs-CRP had a right-skewed distribution, its values were naturally log-transformed before being included in the multivariable models.

#### 2.1.4. Dietary Intake

Data on total energy intake (kcal/day) and protein intake (g/day) were obtained from the KNHANES and analyzed as continuous variables. Dietary intake was assessed using a 24-h dietary recall test. These variables were then included in the sensitivity analysis.

### 2.2. Statistical Analysis

All analyses accounted for the complex survey design of the KNHANES, including the sampling weights, strata, and primary sampling units. Continuous variables are presented as weighted means with standard errors, and categorical variables as unweighted frequencies with weighted percentages. Group differences were examined using complex-samples general linear models or design-adjusted chi-square tests, as appropriate.

Complex-sample logistic regression was used for low muscle strength, and complex sample general linear models were used for maximum handgrip strength. Participants with neither kidney dysfunction nor anemia served as the reference group. Three sequential models were examined: Model 1, adjusted for age and sex; Model 2, the primary confounder-adjusted model, additionally adjusted for diabetes mellitus, hypertension, and myocardial infarction or angina; and Model 3, additionally adjusted for BMI, aerobic physical activity, and log-transformed hs-CRP. Covariates were selected based on clinical knowledge and a causal framework, with the latter three variables treated as potential intermediate or downstream factors. To directly compare the concurrent-condition group with the two single-condition groups, two Model 3 pairwise contrasts were estimated for each outcome, with Bonferroni correction applied to the two contrasts within each outcome. The multiplicative interaction between kidney dysfunction and anemia was assessed in Model 3, and additive interactions for low muscle strength were explored using the relative excess risk due to interaction (RERI), attributable proportion due to interaction (AP), and synergy index, with 95% CIs estimated using the delta method. Effect modification by sex was also tested, and sex-stratified and sex-standardized analyses were performed for maximum handgrip strength.

Missingness was summarized among all 4680 eligible participants, and the included and excluded participants were compared using complex sample models. For missing data sensitivity analysis, the inverse probability of complete-case weighting (IPCW) was applied using the predicted complete-case probabilities among participants with observed outcome and exposure variables, and the resulting weights were combined with the original KNHANES examination weights. Additional sensitivity analyses separately defined kidney dysfunction as a reduced eGFR and albuminuria. Model 3 was also repeated with additional adjustments for total energy and protein intakes among participants with complete nutritional data.

All analyses were performed using IBM SPSS Statistics (version 29; IBM Corp., Armonk, NY, USA) with a complex sample module. All tests were two-sided, and *p* < 0.05 indicated statistical significance.

## 3. Results

### 3.1. Participant Characteristics

A total of 3649 participants were included in the analysis. Table 1 and Table 2 present the weighted sociodemographic, clinical, anthropometric, laboratory, and muscle strength characteristics according to the joint categories of kidney dysfunction and anemia. Significant differences were observed among the four groups for all examined characteristics.

Participants with concurrent kidney dysfunction and anemia were older and had the least favorable renal and muscle strength profiles, including the lowest mean eGFR, maximum handgrip strength, and the highest UACR. The kidney dysfunction-only group showed the highest prevalence of diabetes mellitus and hypertension. The weighted prevalence of low muscle strength was the highest in the concurrent-condition group (27.2%) and lowest in the reference group (3.8%).

### 3.2. Associations of Kidney Dysfunction and Anemia with Low Muscle Strength

In the primary confounder-adjusted model, participants with anemia only had higher odds of low muscle strength than did those with neither condition (adjusted odds ratio [aOR], 2.38; 95% CI, 1.57–3.61), as did participants with concurrent kidney dysfunction and anemia (aOR, 3.31; 95% CI, 1.86–5.89), whereas kidney dysfunction only was not significantly associated with low muscle strength (Table 3). Additional adjustments for BMI, aerobic physical activity, and hs-CRP levels yielded a similar pattern. In direct Model 3 contrasts, the concurrent-condition group had significantly higher odds than the kidney dysfunction-only group (aOR, 2.46; 95% CI, 1.15–5.26; Bonferroni-adjusted *p* = 0.040), whereas the difference with the anemia-only group was not statistically significant after correction (*p* = 0.428) (Table S2). The multiplicative interaction was not statistically significant (*p* = 0.774), and additive interaction estimates were imprecise (RERI, 0.83 [95% CI, −1.40 to 3.05]; AP, 0.25 [95% CI, −0.31 to 0.80]; synergy index, 1.54 [95% CI, 0.50–4.76]).

### 3.3. Adjusted Mean Maximum Handgrip Strength

In the primary confounder-adjusted model, maximum handgrip strength was lower in the anemia-only group (mean difference, −1.36 kg; *p* < 0.001) and the concurrent kidney dysfunction and anemia group (−2.51 kg; *p* < 0.001) than in the reference group, whereas the comparison with the kidney dysfunction-only group was not statistically significant (−0.73 kg; *p* = 0.051) (Table 4). Additional adjustments for BMI, aerobic physical activity, and hs-CRP levels yielded a similar pattern.

In direct Model 3 contrasts, the concurrent group had lower maximum handgrip strength than the kidney dysfunction-only group (mean difference, −1.68 kg; 95% CI, −2.97 to −0.39; Bonferroni-adjusted *p* = 0.022), whereas the difference with the anemia-only group was not statistically significant after correction (*p* = 0.092) (Table S2).

The multiplicative interaction between kidney dysfunction and anemia was not significant (*p* = 0.415).

### 3.4. Sensitivity Analyses

Among the 4680 eligible participants, 1031 (22%) had missing data for at least one analytical variable. Although the excluded participants differed from the included participants in several characteristics, the IPCW-weighted estimates were broadly similar to the complete-case results (Tables S3 and S4); for the kidney dysfunction-only group, the adjusted mean difference in maximum handgrip strength changed only slightly from −0.67 kg (*p* = 0.059) in the complete-case Model 3 analysis to −0.69 kg (*p* = 0.049) in the IPCW Model 3 analysis.

Sensitivity analyses defining kidney dysfunction separately by reduced eGFR and albuminuria showed a generally similar pattern, with the concurrent anemia groups showing unfavorable muscle strength outcomes and weaker or less consistent associations with kidney dysfunction alone (Table S5). Albuminuria only was the most common kidney dysfunction phenotype. In the sensitivity analyses restricted to 3627 participants with complete dietary data, additional adjustments for total energy and protein intakes modestly attenuated the estimates; however, the overall pattern remained unchanged (Table S1).

The kidney dysfunction–anemia category × sex interaction was not significant for low muscle strength (*p* = 0.916) but was significant for maximum handgrip strength (*p* < 0.001). Sex-stratified and sex-standardized analyses supported the overall pattern, with the concurrent group showing the least favorable muscle strength profile (Table S6).

## 4. Discussion

Using data from the nationally representative 2024 KNHANES, this study found poorer muscle strength outcomes in the anemia-only and concurrent kidney dysfunction–anemia groups than in the reference group, whereas the association with kidney dysfunction alone was weaker. The overall pattern remained similar across additional adjustment and sensitivity analyses. Direct comparisons further showed that the concurrent group had poorer muscle strength outcomes than the kidney dysfunction-only group, whereas the differences with the anemia-only group were not statistically significant after Bonferroni correction.

Previous population-based evidence generally supports the relationship among kidney dysfunction, anemia, and impaired physical function, although most studies have examined these factors separately. Farag et al. [25] reported that among adults with CKD stages 3–5, those with anemia had physical function scores approximately 4 points worse than did those without anemia and showed lower physical activity. Cheng et al. [26] found that moderate and high handgrip strengths were associated with approximately 36% and 63% lower odds of CKD, respectively. An earlier analysis of the KNHANES similarly reported an association between low handgrip strength and CKD among Korean adults [27], whereas anemia became increasingly common with greater CKD severity [28]. In the present study, anemia alone was associated with approximately 2.4-fold higher odds of low muscle strength, whereas the concurrent kidney dysfunction and anemia group showed an approximately 3.3-fold increase. These findings extend previous evidence by simultaneously evaluating kidney dysfunction and anemia and distinguishing the concurrent group from each single-condition group.

Evidence from established CKD populations provides a biologically plausible context for this association. CKD-related inflammation, metabolic disturbances, and altered inter-organ signaling may contribute to muscle protein catabolism, impaired anabolic responses, and reduced muscle function [29,30]. Longitudinal evidence also suggests that declining muscle strength during CKD progression may occur independently of changes in skeletal muscle mass [31]. Neuromuscular fatigability and impaired neural drive have also been described in patients with CKD [32,33], indicating that impaired muscle function may reflect not only muscle quantity but also muscle quality and neuromuscular activation. Anemia may add a different but potentially overlapping functional burden. Reduced hemoglobin concentration can limit oxygen-carrying capacity and may contribute to fatigue and reduced exercise tolerance [34]. Furthermore, hemoglobin and handgrip strength have been considered markers related to nutritional and functional status in patients with non-dialysis-dependent CKD [35]. These mechanisms were not directly measured in the present study and should therefore be regarded as potential explanations rather than as demonstrated pathways.

Direct comparisons further refined the interpretation of the four group patterns. After the Bonferroni correction, the concurrent group had significantly higher odds of low muscle strength and lower maximum handgrip strength than had the kidney dysfunction-only group. In contrast, the differences between the concurrent and anemia-only groups were not statistically significant after correction for either outcome. Thus, although the concurrent group had the most unfavorable point estimates, the findings do not support the conclusion that it had significantly poorer muscle strength than both single-condition groups. Accordingly, the findings should be interpreted as indicating a comparatively unfavorable profile rather than demonstrating that anemia necessarily adds to further decline among adults with kidney dysfunction. Neither the multiplicative interaction nor exploratory additive interaction measures provided statistically significant evidence for the interaction. However, the concurrent group was small and the additive interaction estimates were imprecise; therefore, these findings should not be interpreted as evidence that the interaction was absent [36,37].

The findings from the binary and continuous outcomes were generally complementary, while also highlighting the importance of sex in the interpretation of absolute handgrip strength. The sex-specific criterion for low muscle strength identified participants below a clinically recognized threshold, whereas continuous maximum handgrip strength captured differences across the full distribution. Sex significantly modified the association with absolute maximum handgrip strength, whereas no significant effect modification was observed for sex-specific low muscle strength. Nevertheless, the sensitivity analysis using sex-standardized handgrip strength showed a pattern consistent with the primary findings, suggesting that the overall four-group pattern was not solely explained by physiological differences in absolute grip strength between men and women.

Nutritional status may contribute to muscle strength in adults with kidney dysfunction. Higher handgrip strength has been associated with a lower probability of undernutrition in patients with non-dialysis-dependent CKD [38]. In the present study, additional adjustments for total energy and protein intake did not materially change the estimates. This supports the stability of the primary pattern after accounting for these measured nutritional variables, although a single 24-h dietary recall may not adequately represent habitual intake because of day-to-day variations and measurement errors [39]. More broadly, patient-relevant outcomes in CKD include fatigue and physical functioning in addition to laboratory and disease progression measures [40]. Handgrip strength is an objective and readily obtainable measure of muscle function and may provide complementary functional information; however, the present study did not evaluate its screening, predictive, or prognostic performance, and no specific assessment or intervention strategy could be inferred from these findings.

Phenotype-specific sensitivity analyses using reduced eGFR and albuminuria separately showed broadly similar overall patterns, although the associations with kidney dysfunction alone were weaker and less consistent across the phenotypes. Smaller phenotype-specific subgroups limited the precision of estimates and precluded further meaningful analyses based on the severity of kidney dysfunction.

This study has some limitations. First, the cross-sectional design did not allow the temporal sequence of kidney dysfunction, anemia, and low muscle strength to be established, and the observed associations may partly reflect shared underlying health conditions. Second, kidney dysfunction was classified using the eGFR and UACR measured during a single examination. Because clinical CKD requires evidence of persistent abnormalities for at least 3 months, this classification did not correspond to a confirmed diagnosis of CKD. In addition, combining reduced eGFR and albuminuria into a single category limited the assessment of differences according to the type and severity of kidney abnormalities. Since creatinine-based eGFR may be influenced by muscle mass, some misclassifications of kidney dysfunction may also be possible. Phenotype-specific sensitivity analyses using reduced eGFR and albuminuria separately showed broadly similar overall patterns, although the small phenotype-specific subgroups precluded further analysis based on the severity of kidney dysfunction. Third, anemia was defined by hemoglobin concentration, whereas information on ferritin, transferrin saturation, vitamin B12, folate, bleeding, and erythropoiesis-related treatments was unavailable, limiting the characterization of the anemia etiology. Fourth, the concurrent kidney dysfunction and anemia group had a relatively small, unweighted sample size (*n* = 73), which may have reduced the precision of the estimates and their ability to detect interactions. Although neither the multiplicative interaction nor the exploratory additive interaction measures provided statistically significant evidence of interaction, the estimates were imprecise; therefore, these findings should not be interpreted as evidence that interaction was absent. Fifth, 22% of eligible participants were excluded because of missing analytical data, and several characteristics differed between the included and excluded participants. Although the IPCW sensitivity analyses yielded broadly similar estimates, selection bias related to missing data could not be completely excluded. Sixth, residual confounding factors may have remained because information on the cause and severity of kidney disease, metabolic acidosis, neurological disorders, pain, medication use, muscle mass, and other nutritional factors was not fully incorporated. Seventh, dietary intake was assessed using a single 24-h recall and may not represent habitual energy and protein intake. Eighth, handgrip strength measures muscle strength but does not assess muscle mass, gait speed, or other dimensions of physical performance; accordingly, the findings relate specifically to muscle strength rather than to sarcopenia or overall physical functioning. Finally, the study included community-dwelling Korean adults aged ≥40 years, and generalizability to other countries, age groups, institutionalized populations, and patients with advanced CKD may be limited.

## 5. Conclusions

Among Korean adults aged ≥40 years, the concurrent kidney dysfunction and anemia group had the highest estimated odds of low muscle strength and the lowest adjusted mean maximum handgrip strength relative to the participants with neither condition. Anemia alone was also associated with poorer muscle strength outcomes, whereas kidney dysfunction alone was not significantly associated with either outcome in the primary confounder-adjusted model. In direct comparisons, the concurrent group had poorer muscle strength outcomes than the kidney dysfunction-only group, whereas the differences from the anemia-only group were not statistically significant after the Bonferroni correction. Interaction analyses did not provide statistically significant evidence of an interaction, although precision was limited, and the overall pattern remained stable after additional adjustment for total energy and protein intake. These findings suggest that concurrent kidney dysfunction and anemia may indicate a comparatively unfavorable muscle strength profile and warrant further investigation in relation to functional status.

## Figures and Tables

**Table 1:** Weighted participant characteristics stratified by the joint categories of kidney dysfunction and anemia.

Characteristics	No Kidney Dysfunction, No Anemia	Anemia Only	Kidney Dysfunction Only	Kidney Dysfunction + Anemia	*p*
	(*n* = 2956)	(*n* = 329)	(*n* = 291)	(*n* = 73)	
Age, years	57.6 ± 0.35	58.7 ± 0.83	64.6 ± 0.83	69.7 ± 1.76	<0.001
Sex					<0.001
Male	50.9	23.6	56.5	41.2	
Female	49.1	76.4	43.5	58.8	
Aerobic physical activity					<0.001
Yes	41.5	42.5	28.8	32.2	
No	58.5	57.5	71.2	67.8	
Diabetes mellitus					<0.001
Yes	16.8	18.9	46.7	43.7	
No	83.2	81.1	53.3	56.3	
Hypertension					<0.001
Yes	37.2	34.0	78.4	73.6	
No	62.8	66.0	21.6	26.4	
MI or angina					<0.001
Yes	3.1	2.9	7.6	10.7	
No	96.9	97.1	92.4	89.3	

Data are presented as weighted mean ± standard error or weighted percentages. The sample size is unweighted. MI: myocardial infarction.

**Table 2:** Weighted anthropometric, laboratory, and muscle strength measurements according to the joint categories of kidney dysfunction and anemia.

Characteristics	No Kidney Dysfunction + No Anemia	Anemia Only	Kidney Dysfunction Only	Kidney Dysfunction + Anemia	*p*
	(*n* = 2956)	(*n* = 329)	(*n* = 291)	(*n* = 73)	
Body mass index, kg/m^2^	24.3 ± 0.07	23.2 ± 0.25	25.3 ± 0.26	24.1 ± 0.46	<0.001
Hemoglobin, g/dL	14.2 ± 0.03	11.3 ± 0.06	14.3 ± 0.07	11.3 ± 0.13	<0.001
eGFR, mL/min/1.73 m^2^	95.1 ± 0.31	95.6 ± 0.82	80.8 ± 1.51	65.2 ± 3.72	<0.001
UACR, mg/g	6.2 ± 0.10	7.1 ± 0.30	114.3 ± 13.00	190.7 ± 74.68	<0.001
hs-CRP, mg/L	1.3 ± 0.07	1.8 ± 0.45	2.1 ± 0.30	3.5 ± 2.38	0.031
Maximum handgrip strength, kg	33.1 ± 0.23	27.2 ± 0.51	31.4 ± 0.58	25.9 ± 0.87	<0.001
Low muscle strength, %					<0.001
Yes	143 (3.8%)	41 (11.3%)	37 (9.7%)	22 (27.2%)	
No	2813 (96.2%)	288 (88.7%)	254 (90.3%)	51 (72.8%)	

Data are presented as weighted mean ± standard error or unweighted *n* (weighted %). Low muscle strength was defined as a maximum handgrip strength <28 kg and <18 kg in men and women, respectively. eGFR, estimated glomerular filtration rate; hs-CRP, high-sensitivity C-reactive protein; UACR, urinary albumin-to-creatinine ratio.

**Table 3:** Associations of the joint categories of kidney dysfunction and anemia with low muscle strength.

Joint Categories	Unweighted *n* (Cases)	Model 1 aOR (95% CI)	Model 2 aOR (95% CI)	Model 3 aOR (95% CI)
No kidney dysfunction + no anemia	2956 (143)	1.00 (Reference)	1.00 (Reference)	1.00 (Reference)
Anemia only	329 (41)	2.45 (1.60–3.74)	2.38 (1.57–3.61)	2.16 (1.41–3.30)
Kidney dysfunction only	291 (37)	1.41 (0.88–2.25)	1.42 (0.88–2.30)	1.36 (0.82–2.25)
Kidney dysfunction + anemia	73 (22)	3.33 (1.90–5.86)	3.31 (1.86–5.89)	3.35 (1.88–5.95)

Unweighted sample sizes are presented as total *n* (*n* with low muscle strength). Model 1 was adjusted for age and sex; Model 2 was adjusted for age, sex, diabetes mellitus, hypertension, and myocardial infarction or angina; and Model 3 included the Model 2 covariates and additionally adjusted for body mass index, aerobic physical activity, and log-transformed hs-CRP. aOR: adjusted odds ratio; CI: confidence interval; hs-CRP: high-sensitivity C-reactive protein.

**Table 4:** Adjusted maximum handgrip strength according to the joint categories of kidney dysfunction and anemia.

Joint Category	Model 1, kg (95% CI)	Model 2, kg (95% CI)	Model 3, kg (95% CI)	Difference, kg (SE)	*p*
No kidney dysfunction + no anemia	32.72 (32.49–32.95)	32.10 (31.59–32.61)	31.78 (31.27–32.30)	Reference	
Anemia only	31.31 (30.63–32.00)	30.74 (29.94–31.54)	30.69 (29.87–31.51)	−1.36 (0.34)	<0.001
Kidney dysfunction only	32.05 (31.39–32.72)	31.38 (30.54–32.21)	31.12 (30.31–31.92)	−0.73 (0.37)	0.051
Kidney dysfunction + anemia	30.20 (28.99–31.41)	29.59 (28.33–30.85)	29.44 (28.23–30.64)	−2.51 (0.61)	<0.001

Model 1 was adjusted for age and sex; Model 2 was adjusted for age, sex, diabetes mellitus, hypertension, and myocardial infarction or angina; Model 3 included the Model 2 covariates and additionally adjusted for body mass index, aerobic physical activity, and log-transformed hs-CRP. Differences were estimated from Model 2, using participants with no kidney dysfunction and no anemia as the reference group. CI, confidence interval; SE, standard error.

## Data Availability

The data analyzed in this study are available from the Korea National Health and Nutrition Examination Survey (KNHANES) website of the Korea Disease Control and Prevention Agency (https://knhanes.kdca.go.kr/knhanes/ (accessed on 6 August 2026)) upon completion of the required data use procedures. No new data were obtained in this study.

## Supplementary Materials

The following supporting information can be downloaded at: https://www.mdpi.com/article/10.3390/healthcare14183043/s1, Table S1: Sensitivity analysis of low muscle strength after additional adjustment for total energy and protein intake; Table S2: Direct Model 3 pairwise contrasts comparing the concurrent kidney dysfunction–anemia group with single-condition groups; Table S3: Missing-data assessment among eligible participants aged ≥ 40 years; Table S4: Inverse probability of complete-case weighting sensitivity analyses; Table S5: Kidney dysfunction phenotype distribution and phenotype-specific sensitivity analyses; and Table S6: Sex-related interaction, stratified, and standardized handgrip-strength analyses.

## Abbreviations

The following abbreviations are used in this manuscript:

aOR	adjusted odds ratio
AP	attributable proportion due to interaction
BMI	body mass index
CKD	chronic kidney disease
CI	confidence interval
eGFR	estimated glomerular filtration rate
hs-CRP	high-sensitivity C-reactive protein
IPCW	inverse probability of complete-case weighting
KNHANES	Korea National Health and Nutrition Examination Survey
MI	myocardial infarction
OR	odds ratio
RERI	relative excess risk due to interaction
SE	standard error
HR	hazard ratio
UACR	urinary albumin-to-creatinine ratio

## References

[1] Cruz-Jentoft A.J., Bahat G., Bauer J., Boirie Y., Bruyère O., Cederholm T., Cooper C., Landi F., Rolland Y., Sayer A.A. (2019). Sarcopenia: Revised European consensus on definition and diagnosis. Age Ageing.

[2] Chen L.-K., Woo J., Assantachai P., Auyeung T.-W., Chou M.-Y., Iijima K., Jang H.C., Kang L., Kim M., Kim S. (2020). Asian working group for sarcopenia: 2019 consensus update on sarcopenia diagnosis and treatment. J. Am. Med. Dir. Assoc..

[3] Ribeiro L.W., Berndt S., Mielke G.I., Doust J., Mishra G.D. (2024). Factors associated with handgrip strength across the life course: A systematic review. J. Cachexia Sarcopenia Muscle.

[4] López-Bueno R., Andersen L.L., Koyanagi A., Núñez-Cortés R., Calatayud J., Casaña J., del Pozo Cruz B. (2022). Thresholds of handgrip strength for all-cause, cancer, and cardiovascular mortality: A systematic review with dose-response meta-analysis. Ageing Res. Rev..

[5] He P., Ye Z., Liu M., Li H., Zhang Y., Zhou C., Wu Q., Zhang Y., Yang S., Liu C. (2023). Association of handgrip strength and/or walking pace with incident chronic kidney disease: A UK Biobank observational study. J. Cachexia Sarcopenia Muscle.

[6] Yan L., Hu X., Wu S., Chen L., Zhao S. (2024). Association between grip strength and albuminuria in the general United States population: NHANES 2011–2014. Front. Public Health.

[7] Duarte M.P., Almeida L.S., Neri S.G., Oliveira J.S., Wilkinson T.J., Ribeiro H.S., Lima R.M. (2024). Prevalence of sarcopenia in patients with chronic kidney disease: A global systematic review and meta-analysis. J. Cachexia Sarcopenia Muscle.

[8] Ribeiro H.S., Neri S.G., Oliveira J.S., Bennett P.N., Viana J.L., Lima R.M. (2022). Association between sarcopenia and clinical outcomes in chronic kidney disease patients: A systematic review and meta-analysis. Clin. Nutr..

[9] Zhang F., Wang H., Bai Y., Huang L., Zhang H. (2023). Handgrip strength and all-cause mortality in patients with chronic kidney disease: An updated systematic review and meta-analysis of cohort studies. Int. Urol. Nephrol..

[10] Chen H., Zhang F., Huang L., Bai Y., Zhong Y., Li Y. (2024). Thresholds of handgrip strength for all-cause mortality in patients with chronic kidney disease: A secondary systematic review with dose-response meta-analysis. Ren. Fail..

[11] Kim D.W., Song S.H. (2023). Sarcopenia in chronic kidney disease: From bench to bedside. Korean J. Intern. Med..

[12] Bakinowska E., Olejnik-Wojciechowska J., Kiełbowski K., Skoryk A., Pawlik A. (2024). Pathogenesis of sarcopenia in chronic kidney disease—The role of inflammation, metabolic dysregulation, gut dysbiosis, and microRNA. Int. J. Mol. Sci..

[13] Badura K., Janc J., Wąsik J., Gnitecki S., Skwira S., Młynarska E., Rysz J., Franczyk B. (2024). Anemia of chronic kidney disease—A narrative review of its pathophysiology, diagnosis, and management. Biomedicines.

[14] Lee D.Y., Shin S. (2023). Sarcopenia and anemia in elderly koreans: A nationwide population-based study. Healthcare.

[15] Lee B.J., Chi J.H. (2023). Association between anemia and grip strength indices combined with anthropometry in the Korean population. Sci. Rep..

[16] Hammer T., Braisch U., Rothenbacher D., Denkinger M., Dallmeier D. (2024). Relationship between hemoglobin and grip strength in older adults: The ActiFE study. Aging Clin. Exp. Res..

[17] Wang H., Lin P. (2024). Association between sarcopenia and hemoglobin level: A systematic review and meta-analysis. Front. Med..

[18] De La Cruz-Góngora V., Salinas-Rodriguez A., Manrique-Espinoza B. (2024). Prospective changes in anemia are associated with the incidence and persistence of sarcopenia among older Mexican adults. Front. Nutr..

[19] Inker L.A., Eneanya N.D., Coresh J., Tighiouart H., Wang D., Sang Y., Crews D.C., Doria A., Estrella M.M., Froissart M. (2021). New creatinine- and cystatin C–based equations to estimate GFR without race. N. Engl. J. Med..

[20] Levin A., Ahmed S.B., Carrero J.J., Foster B., Francis A., Hall R.K., Herrington W.G., Hill G., Inker L.A., Kazancıoğlu R. (2024). Executive summary of the KDIGO 2024 Clinical Practice Guideline for the Evaluation and Management of Chronic Kidney Disease: Known knowns and known unknowns. Kidney Int..

[21] World Health Organization (2024). Guideline on Haemoglobin Cutoffs to Define Anaemia in Individuals and Populations.

[22] Bull F.C., Al-Ansari S.S., Biddle S., Borodulin K., Buman M.P., Cardon G., Carty C., Chaput J.-P., Chastin S., Chou R. (2020). World Health Organization 2020 guidelines on physical activity and sedentary behaviour. Br. J. Sports Med..

[23] Hur K.Y., Moon M.K., Park J.S., Kim S.-K., Lee S.-H., Yun J.-S., Baek J.H., Noh J., Lee B.-W., Oh T.J. (2021). 2021 clinical practice guidelines for diabetes mellitus of the Korean Diabetes Association. Diabetes Metab. J..

[24] Kim H.L., Lee E.M., Ahn S.Y., Kim K.I., Kim H.C., Kim J.H., Lee H.Y., Lee J.H., Park J.M., Cho E.J. (2023). The 2022 focused update of the 2018 Korean hypertension Society Guidelines for the management of hypertension. Clin. Hypertens..

[25] Farag Y.M., Blasco-Colmenares E., Zhao D., Sanon M., Guallar E., Finkelstein F.O. (2023). Effect of anemia on physical function and physical activity in CKD: The National Health and Nutrition Examination Survey, 1999–2016. Kidney360.

[26] Cheng Y., Liu M., Liu Y., Xu H., Chen X., Zheng H., Wu X., Shen Z., Shen C. (2021). Chronic kidney disease: Prevalence and association with handgrip strength in a cross-sectional study. BMC Nephrol..

[27] Lee Y.L., Jin H., Lim J.-Y., Lee S.Y. (2021). Relationship between low handgrip strength and chronic kidney disease: KNHANES 2014–2017. J. Ren. Nutr..

[28] Kovesdy C.P., Davis J.R., Duling I., Little D.J. (2023). Prevalence of anaemia in adults with chronic kidney disease in a representative sample of the United States population: Analysis of the 1999–2018 National Health and Nutrition Examination Survey. Clin. Kidney J..

[29] Wang X.H., Price S.R. (2023). Organ crosstalk contributes to muscle wasting in chronic kidney disease. Semin. Nephrol..

[30] Price S.R., Wang X.H. (2025). Protein-energy wasting in chronic kidney disease: Mechanisms responsible for loss of muscle mass and function. Kidney Res. Clin. Pract..

[31] Chatrenet A., de Müllenheim P.-Y., Torreggiani M., Hernández J.N., Arronte R.U., Espinoza A.G., Piccoli G.B. (2025). Quality matters: Chronic kidney disease progression is associated with reduced muscle strength independently of changes in skeletal muscle mass: An observational study. Clin. Kidney J..

[32] Chatrenet A., Piccoli G., Audebrand J.M., Torreggiani M., Barbieux J., Vaillant C., Morel B., Durand S., Beaune B. (2023). Analysis of the rate of force development reveals high neuromuscular fatigability in elderly patients with chronic kidney disease. J. Cachexia Sarcopenia Muscle.

[33] Chatrenet A., Piccoli G., Anthierens A., Torreggiani M., Audebrand J.M., Morel B., Beaune B., Durand S. (2022). Neural drive impairment in chronic kidney disease patients is associated with neuromuscular fatigability and fatigue. Med. Sci. Sports Exerc..

[34] Auerbach M., DeLoughery T.G., Tirnauer J.S. (2025). Iron deficiency in adults: A review. JAMA.

[35] Lonardo M.S., Cacciapuoti N., Chiurazzi M., Di Lauro M., Guida B., Damiano S., Cataldi M. (2023). Combined use of handgrip strength and hemoglobin as markers of undernutrition in patients with stage 3–5 chronic kidney disease. Nutr. Metab. Cardiovasc. Dis..

[36] Knol M.J., VanderWeele T.J. (2012). Recommendations for presenting analyses of effect modification and interaction. Int. J. Epidemiol..

[37] Knol M.J., VanderWeele T.J., Groenwold R.H., Klungel O.H., Rovers M.M., Grobbee D.E. (2011). Estimating measures of interaction on an additive scale for preventive exposures. Eur. J. Epidemiol..

[38] Kim M., Park Y.W., Im D.W., Jeong Y., Noh H.J., Yang S.J., Kang E., Ryu H., Kim J., Koo J.R. (2024). Association of handgrip strength and nutritional status in non-dialysis-dependent chronic kidney disease patients: Results from the KNOW-CKD study. Nutrients.

[39] Gibson R.S., Charrondiere U.R., Bell W. (2017). Measurement errors in dietary assessment using self-reported 24-hour recalls in low-income countries and strategies for their prevention. Adv. Nutr..

[40] Parra E., Salgueira M., Portolés J., Serrano P., Bayés B., Estévez J., Del Pino M.D. (2023). Standardizing health outcomes for chronic kidney disease. Adaptation of the international consortium for health outcomes measurement standard set to the Spanish setting. Nefrologia.

