# Supplementary material for: Muscle Strength Profiles According to Kidney Dysfunction and Anemia Status in Korean Adults Aged ≥40 Years: A Nationwide Cross-Sectional Study
Source: Healthcare (Basel). 2026 Sep 16;14(18):3043. doi: 10.3390/healthcare14183043 (PMC13606201; doi:10.3390/healthcare14183043)
Supplement: Supplementary file 1 [file healthcare-14-03043-s001.zip › healthcare-4542080-supplementary.pdf]

Supplementary tables

Table S1. Sensitivity analysis of low muscle strength after additional adjustment for total energy and protein intake.

Conjoint category	Model 3 aOR (95% CI)	Model 3 + total energy and protein intake aOR (95% CI)
No kidney dysfunction + no anemia	1.00 (Reference)	1.00 (Reference)
Anemia only	2.07 (1.36–3.17)	1.94 (1.27–2.97)
Kidney dysfunction only	1.37 (0.83–2.25)	1.31 (0.80–2.16)
Kidney dysfunction + anemia	3.27 (1.81–5.90)	3.03 (1.65–5.56)

Values are adjusted odds ratios (aORs) with 95% confidence intervals (CIs). Both models included age, sex, body mass index, diabetes mellitus, hypertension, myocardial infarction or angina, aerobic physical activity, and log-transformed high-sensitivity C-reactive protein. The second model additionally included total energy and protein intake. Analyses were restricted to participants with complete nutrition data (n = 3,627).

Table S2. Direct Model 3 pairwise contrasts comparing the concurrent kidney dysfunction–anemia group with single-condition groups.

Outcome	Comparison	Adjusted estimate	95% CI	Raw *p*	Bonferroni-adjusted *p*
Low muscle strength	Concurrent vs. anemia only	aOR 1.55	0.78–3.11	0.214	0.428
Low muscle strength	Concurrent vs. kidney dysfunction only	aOR 2.46	1.15–5.26	0.020	0.040
Maximum handgrip strength	Concurrent vs. anemia only	−1.26 kg	−2.49 to −0.02	0.046	0.092
Maximum handgrip strength	Concurrent vs. kidney dysfunction only	−1.68 kg	−2.97 to −0.39	0.011	0.022

Estimates were obtained from Model 3, adjusted for age, sex, diabetes mellitus, hypertension, myocardial infarction or angina, body mass index, aerobic physical activity, and log-transformed high-sensitivity C-reactive protein, with the KNHANES complex survey design incorporated. Bonferroni correction was applied to the two direct contrasts within each outcome. For maximum handgrip strength, negative values indicate lower strength in the concurrent group. The 95% CIs are conventional model-based intervals. aOR, adjusted odds ratio; CI, confidence interval; KNHANES, Korea National Health and Nutrition Examination Survey.

Table S3. Missing-data assessment among eligible participants aged ≥40 years.

Panel A. Variable-specific missingness (N = 4,680)

Variables	Valid n	Missing n	Missing, %
Maximum handgrip strength	4,180	500	10.7
eGFR	4,502	178	3.8
UACR	4,492	188	4.0
Hemoglobin	4,498	182	3.9
Body mass index	4,578	102	2.2
Aerobic physical activity	4,214	466	10.0
Diabetes mellitus	4,354	326	7.0
Hypertension	4,613	67	1.4
Myocardial infarction or angina	4,223	457	9.8
hs-CRP	4,502	178	3.8
Sex	4,680	0	0.0
Age	4,680	0	0.0

Missingness could overlap across variables; therefore, variable-specific missing counts should not be summed. Overall, 1,031 participants (22.0%) had missing data for at least one analytic variable and 3,649 (78.0%) were included in the complete-case analysis. eGFR, estimated glomerular filtration rate; hs-CRP, high-sensitivity C-reactive protein; UACR, urinary albumin-to-creatinine ratio.

Panel B. Comparison of participants included in and excluded from the complete-case analysis

Characteristic	Excluded (n = 1,031)	Included (n = 3,649)	*p*
Age, years	60.92 (0.61)	58.37 (0.35)	< 0.001
Sex			0.155
Male, n (weighted %)	420 (46.4)	1,547 (48.9)	
Female, n (weighted %)	611 (53.6)	2,102 (51.1)	
Body mass index, kg/m²	24.44 (0.14)	24.32 (0.07)	0.451
Hemoglobin, g/dL	13.79 (0.06)	13.95 (0.03)	0.016

eGFR, mL/min/1.73 m^2	92.08 (0.75)	93.59 (0.33)	0.051
ln(UACR)	2.098 (0.042)	1.822 (0.022)	< 0.001
ln(hs-CRP)	−0.416 (0.041)	−0.573 (0.020)	0.001
Diabetes mellitus, yes, n (weighted %)	170 (21.8)	760 (19.7)	0.278
Hypertension, yes, n (weighted %)	483 (46.1)	1,581 (40.6)	0.006
Myocardial infarction or angina, yes, n (weighted %)	28 (4.6)	154 (3.5)	0.220
Aerobic physical activity, yes, n (weighted %)	188 (34.8)	1,416 (40.5)	0.045

Continuous variables are presented as survey-weighted mean (standard error), and categorical variables as unweighted n (survey-weighted %). p-values were obtained using complex-samples general linear models for continuous variables and complex-samples logistic regression for categorical variables. Comparisons used available observations for each characteristic; therefore, denominators among excluded participants varied by variable. ln, natural logarithm.

Table S4. Inverse probability of complete-case weighting sensitivity analyses

Panel A. Low muscle strength

Conjoint category	Complete-case Model 3, aOR (95% CI)	IPCW Model 3, aOR (95% CI)
No kidney dysfunction + no anemia	1.00 (Reference)	1.00 (Reference)
Anemia only	2.16 (1.41–3.30)	2.15 (1.41–3.27)
Kidney dysfunction only	1.36 (0.82–2.25)	1.34 (0.81–2.20)
Kidney dysfunction + anemia	3.35 (1.88–5.95)	3.33 (1.87–5.96)

Panel B. Maximum handgrip strength

Conjoint category	Complete-case adjusted mean, kg (95% CI)	IPCW adjusted mean, kg (95% CI)	Complete-case difference vs. reference, kg (p)	IPCW difference vs. reference, kg (p)
No kidney dysfunction + no anemia	31.78 (31.27–32.30)	31.67 (31.15–32.18)	Reference	Reference
Anemia only	30.69 (29.87–31.51)	30.53 (29.72–31.35)	−1.09 (0.002)	−1.13 (0.001)
Kidney dysfunction only	31.12 (30.31–31.92)	30.98 (30.18–31.78)	−0.67 (0.059)	−0.69 (0.049)
Kidney dysfunction + anemia	29.44 (28.23–30.64)	29.23 (28.01–30.46)	−2.35 (<0.001)	−2.43 (<0.001)

IPCW, inverse probability of complete-case weighting. IPCW analyses used the original KNHANES examination weight multiplied by the inverse probability of complete-case inclusion. Model 3 adjusted for age, sex, diabetes mellitus, hypertension, myocardial infarction or angina, body mass index, aerobic physical activity, and log-transformed high-sensitivity C-reactive protein. CI, confidence interval aOR, adjusted odds ratio; CI, confidence interval; KNHANES, Korea National Health and Nutrition Examination Survey.

Table S5. Kidney dysfunction phenotype distribution and phenotype-specific sensitivity analyses.

Panel A. Distribution of kidney dysfunction phenotypes in the complete-case sample

Kidney dysfunction phenotype	Kidney dysfunction only (n = 291)	Kidney dysfunction + anemia (n = 73)
Reduced eGFR only	56 (19.2%)	26 (35.6%)
Albuminuria only	203 (69.8%)	30 (41.1%)
Reduced eGFR + albuminuria	32 (11.0%)	17 (23.3%)

Panel B. Low muscle strength

Kidney dysfunction definition	Category	aOR (95% CI)
Reduced eGFR	No reduced eGFR + no anemia	1.00 (Reference)
	Anemia only	2.27 (1.54–3.34)
	Reduced eGFR only	1.78 (0.97–3.26)
	Reduced eGFR + anemia	3.01 (1.43–6.34)
Albuminuria	No albuminuria + no anemia	1.00 (Reference)
	Anemia only	2.14 (1.45–3.17)
	Albuminuria only	0.95 (0.52–1.73)
	Albuminuria + anemia	3.13 (1.37–7.13)

Panel C. Maximum handgrip strength

Kidney dysfunction definition	Category	Adjusted mean, kg (95% CI)	Difference vs. reference, kg	*p*
Reduced eGFR	No reduced eGFR + no anemia	31.69 (31.16–32.21)	Reference	
	Anemia only	30.60 (29.81–31.40)	−1.09	0.001
	Reduced eGFR only	31.53 (30.35–32.71)	−0.16	0.795
	Reduced eGFR + anemia	28.96 (27.30–30.62)	−2.73	< 0.001
Albuminuria	No albuminuria + no anemia	31.78 (31.27–32.29)	Reference	
	Anemia only	30.60 (29.79–31.41)	−1.18	< 0.001
	Albuminuria only	31.01 (30.14–31.89)	−0.77	0.043
	Albuminuria + anemia	29.59 (28.14–31.05)	−2.19	0.003

Phenotype-specific models used the complete-case sample (N = 3,649) and were adjusted using Model 3 covariates. Reduced eGFR was defined as eGFR <60 mL/min/1.73 m²; albuminuria was defined as UACR ≥30 mg/g. aOR, adjusted odds ratio; CI, confidence interval; eGFR, estimated glomerular filtration rate; UACR, urinary albumin-to-creatinine ratio.

Table S6. Sex-related interaction, stratified, and standardized handgrip-strength analyses.

Panel A. Effect modification by sex

Outcome	Wald F	df1	df2	*p* for kidney dysfunction–anemia category × sex
Low muscle strength	0.171	3	158	0.916
Maximum handgrip strength	8.759	3	158	<0.001

Panel B. Sex-stratified maximum handgrip strength

Sex	Conjoint category	Adjusted mean, kg (95% CI)	Difference vs. reference, kg	p
Men	No kidney dysfunction + no anemia	39.79 (39.03–40.54)	Reference	
	Anemia only	37.79 (36.27–39.31)	−2.00	0.003
	Kidney dysfunction only	38.60 (37.33–39.86)	−1.19	0.032
	Kidney dysfunction + anemia	36.91 (34.41–39.42)	−2.87	0.025
Women	No kidney dysfunction + no anemia	23.95 (23.17–24.72)	Reference	
	Anemia only	23.55 (22.35–24.75)	−0.40	0.318
	Kidney dysfunction only	24.25 (23.34–25.16)	+0.30	0.437
	Kidney dysfunction + anemia	22.46 (21.25–23.68)	−1.48	0.007

Panel C. Sex-standardized maximum handgrip strength

**Conjoint category**	**Adjusted standardized HGS, z score (95% CI)**	**Difference vs. reference, SD**	**p**
No kidney dysfunction + no anemia	−0.12 (−0.21 to −0.03)	Reference	
Anemia only	−0.31 (−0.47 to −0.16)	−0.20	0.003
Kidney dysfunction only	−0.17 (−0.30 to −0.04)	−0.05	0.389
Kidney dysfunction + anemia	−0.50 (−0.71 to −0.30)	−0.39	<0.001

Sex interaction tests were obtained from Model 3. Sex-stratified models omitted sex as a covariate and otherwise used the Model 3 adjustment set. Sex-standardized handgrip strength was calculated within sex before complex-samples analysis. HGS, handgrip strength; SD, standard deviation; CI, confidence interval; df1 and df2, numerator and denominator degrees of freedom, respectively.
